# Probing electron-hole Coulomb correlations in the exciton landscape of a twisted semiconductor heterostructure

**DOI:** 10.1126/sciadv.adi1323

**Published:** 2024-02-07

**Authors:** Jan Philipp Bange, David Schmitt, Wiebke Bennecke, Giuseppe Meneghini, AbdulAziz AlMutairi, Kenji Watanabe, Takashi Taniguchi, Daniel Steil, Sabine Steil, R. Thomas Weitz, G. S. Matthijs Jansen, Stephan Hofmann, Samuel Brem, Ermin Malic, Marcel Reutzel, Stefan Mathias

**Affiliations:** ^1^ I. Physikalisches Institut, Georg-August-Universität Göttingen, Friedrich-Hund-Platz 1, 37077 Göttingen, Germany.; ^2^ Fachbereich Physik, Philipps-Universität Marburg, 35032 Marburg, Germany.; ^3^ Department of Engineering, University of Cambridge, Cambridge CB3 0FA, UK.; ^4^ Research Center for Functional Materials, National Institute for Materials Science, 1-1 Namiki, Tsukuba 305-0044, Japan.; ^5^ International Center for Materials Nanoarchitectonics, National Institute for Materials Science, 1-1 Namiki, Tsukuba 305-0044, Japan.; ^6^ International Center for Advanced Studies of Energy Conversion (ICASEC), University of Göttingen, Göttingen, Germany.; ^7^ Department of Physics, Chalmers University of Technology, Gothenburg, Sweden.

## Abstract

In two-dimensional semiconductors, cooperative and correlated interactions determine the material’s excitonic properties and can even lead to the creation of correlated states of matter. Here, we study the fundamental two-particle correlated exciton state formed by the Coulomb interaction between single-particle holes and electrons. We find that the ultrafast transfer of an exciton’s hole across a type II band-aligned semiconductor heterostructure leads to an unexpected sub-200-femtosecond upshift of the single-particle energy of the electron being photoemitted from the two-particle exciton state. While energy relaxation usually leads to an energetic downshift of the spectroscopic signature, we show that this upshift is a clear fingerprint of the correlated interaction of the electron and hole parts of the exciton. In this way, time-resolved photoelectron spectroscopy is straightforwardly established as a powerful method to access electron-hole correlations and cooperative behavior in quantum materials. Our work highlights this capability and motivates the future study of optically inaccessible correlated excitonic and electronic states of matter.

## Teaser

Time-resolved photoelectron spectroscopy is directly sensitive to electron-hole Coulomb correlations in a 2D heterostructure.

## INTRODUCTION

An exciton is a prime example of a quasiparticle that is built up by electrons and holes bound together via Coulomb interaction. As in the case of a hydrogen atom, the exciton’s properties are described by its quantum number, its binding energy, and its Bohr radius (*1*). For low-dimensional materials, these key parameters can be substantially altered by cooperative interactions with surrounding quasiparticles (*2*, *3*). To study such cooperative and emergent behavior, artificial stacks of two-dimensional transition metal dichalcogenides (TMDs) have been shown to provide an exceptional playground for manipulating exciton properties. Examples include the ultrafast formation of interlayer excitons whose electron and hole components are charge-separated across the neighboring TMD layers (*4*–*8*), the confinement of excitons in a moiré potential well (*9*–*12*), the creation of correlated interlayer exciton insulators (*13*, *14*) and exciton crystals (*15*, *16*), and even the stabilization of Bose-Einstein condensates (*17*).

**Fig. 1.**
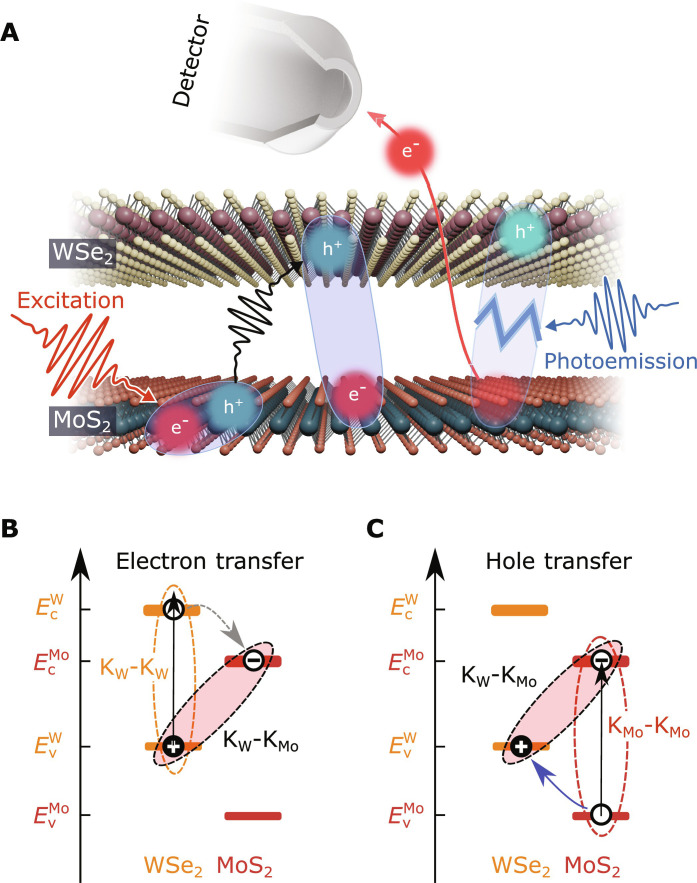
Probing Coulomb-correlated electron-hole pairs and their femtosecond dynamics using momentum microscopy. (**A**) Schematic illustration of the photoemission process from excitons. Visible femtosecond light pulses (red) are used to optically excite bright excitons that fully reside in the MoS_2_ monolayer. The transfer of the hole component into the WSe_2_ monolayer leads to the formation of charge-separated interlayer excitons (black arrow). A time-delayed extreme ultraviolet laser pulse (blue) breaks the exciton; single-particle electrons are detected in the photoelectron analyzer and single-particle holes remain in the WSe_2_ monolayer. (**B** and **C**) Single-particle energy-level alignment of the valence and conduction bands (v and c) of MoS_2_ and WSe_2_. K_W_-K_Mo_ excitons are formed due to interlayer charge transfer of the exciton’s hole or electron, respectively, from intralayer K_Mo_-K_Mo_ or K_W_-K_W_ excitons. Note that in (C), the electron contribution to the exciton remains rigid in the conduction band minimum of MoS_2_ during the hole-transfer process. In the abbreviation of the excitons, the capital letters and the subscripts denote the valley (K, Σ, and Γ) and the layer (W and Mo) where the exciton’s hole (first letter) and electron (second letter) are localized. It is not differentiated between momentum-direct and momentum-indirect excitons (e.g., K_W/Mo_ and K'_W/Mo_ or Σ and Σ') because those cannot be differentiated in the photoemission experiment (see fig. S7).

It is therefore of fundamental importance to obtain insight into the energy landscape and the ultrafast dynamics of the two-particle correlated exciton state (*18*, *19*). In TMD semiconductors, momentum-indirect and spin-forbidden excitons play a substantial role but are mostly inaccessible (*7*, *20*) using all-optical experimental techniques (*21*, *22*). Recently, time- and angle-resolved photoelectron spectroscopy (trARPES) experiments have been shown to be a powerful technique to fill this gap and to simultaneously probe the energy landscape and dynamics of optically bright and dark excitons in monolayer (*23*–*25*) and twisted bilayer (*8*, *12*, *26*, *27*) TMDs. When using photoelectron spectroscopy, there is a fundamental aspect that needs to be considered (Fig. 1A): In the photoemission process, the Coulomb correlation between the electron and hole components of the exciton is broken. This is because a single-particle photoelectron is collected with the detector and a single-particle hole remains in the material (*28*–*31*). In consequence, photoelectrons originating from excitons are detected at the exciton binding energy below the conduction band minimum (*8*, *23*–*25*, *32*) and show a hole-like energy-momentum dispersion (*32*, *33*). In this way, trARPES provides natural access to the electron contribution of the exciton and can be used to quantify the charge transfer of the exciton’s electron across a type II band-aligned heterostructure (Fig. 1B) (*8*, *27*). However, to this day, only very limited energy- and momentum-resolved spectroscopic information on the exciton’s hole component is reported (*12*). Specifically, in contrast to all-optical spectroscopies (*4*–*6*, *18*, *34*–*37*), it has not been shown that trARPES can be applied to monitor the charge-transfer dynamics of the exciton’s hole across the TMD interface (Fig. 1C).

Here, we demonstrate how the Coulomb interaction between the electron- and the hole components of the intra- and interlayer excitons facilitates the study of the ultrafast hole-transfer mechanism in a twisted WSe_2_/MoS_2_ heterostructure. We experimentally observe an increase in the exciton’s photoelectron energy upon the hole-transfer process across the interface. This is unexpected at first because the electron remains rigid in the conduction band minimum during this hole-transfer process (Fig. 1C) and because any relaxation mechanism is typically expected to cause an overall decrease in the measured electronic quasiparticle energies. However, when taking the correlated nature of the electron-hole pair into account, despite an overall decrease in the quasiparticle energies, we show that such an increase due to hole transfer must be expected for the corresponding exciton’s photoelectron. Our work provides microscopic insights into the ultrafast hole-transfer mechanism and, more generally, highlights the potential of time-resolved momentum microscopy to probe optically inaccessible correlated excitonic and electronic states of matter.

## RESULTS

### Energy landscape and photoemission fingerprints of bright and dark excitons

**Fig. 2.**
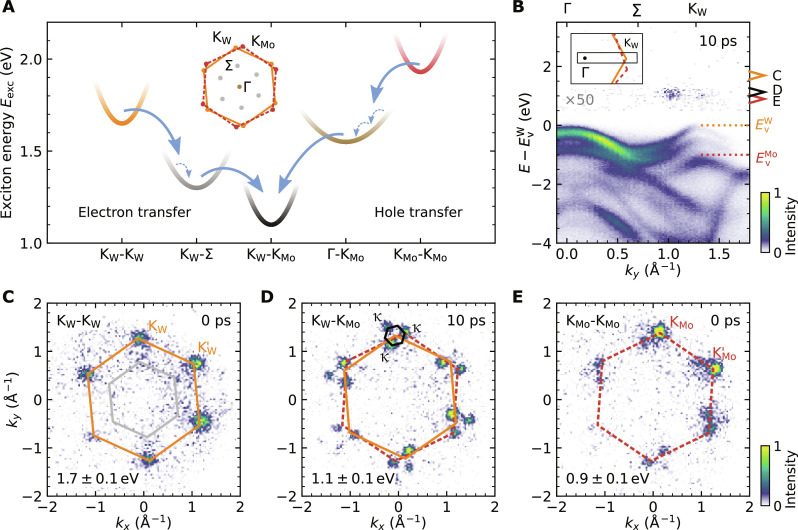
Energy landscape and energy-momentum fingerprints of excitons in WSe_2_/MoS_2_. (**A**) Calculated low-energy exciton landscape of intralayer, hybrid, and interlayer excitons. The electron- and hole-transfer processes can be initiated via excitation with 1.7- and 1.9-eV light pulses, respectively, and proceed via the K_W_-K_W_ → K_W_-Σ→ K_W_-K_Mo_ and K_Mo_-K_Mo_ → Γ-K_Mo_ → K_W_-K_Mo_ cascades. The solid and dashed arrows, respectively, indicate exciton-phonon scattering events leading to inter- and intravalley thermalization of the exciton occupation. The effective mass of the exciton dispersion is extracted from many-body calculations. The inset schematically shows the alignment of the WSe_2_ and MoS_2_ Brillouin zones and indicates the high-symmetry points in the first Brillouin zone. (**B**) Energy- and momentum-resolved photoemission spectrum along the Γ-Σ-K_W_ direction (inset) measured on the WSe_2_/MoS_2_ heterostructure after photoexcitation with 1.9-eV light pulses at a delay of 10 ps. The WSe_2_ and MoS_2_ valence band maxima are labeled with $E_{v}^{W}$ and $E_{v}^{\text{Mo}}$, respectively. (**C** to **E**) Photoemission momentum fingerprints of the (C) intralayer K_W_-K_W_ exciton (0 ps), the (D) interlayer K_W_-K_Mo_ exciton (10 ps), and the (E) intralayer K_Mo_-K_Mo_ exciton (0 ps) after photoexcitation with 1.9-eV light pulses. The photoelectron energies of the momentum maps are given in the figure with respect to the energy of the WSe_2_ valence band maximum and are indicated by colored arrowheads in (B). The energetic width of the arrowheads indicates the energy range used for generating the momentum maps (C, D, and E). The Brillouin zones of WSe_2_, MoS_2_, and the moiré superlattice are overlaid on the data by orange, dark red (dashed), and black hexagons, respectively.

We start the analysis of the hole-transfer dynamics by first calculating the full energy landscape and formation dynamics of bright and dark excitons in the twisted WSe_2_/MoS_2_ heterostructure on a microscopic footing (details in Supplementary Text). The optically excited A1s excitons in the WSe_2_ and the MoS_2_ layer and their cascaded relaxation via layer-hybridized excitons to the lowest energy interlayer excitons are illustrated in Fig. 2A. If the heterostructure is excited resonantly to the A1s-exciton of WSe_2_ with 1.7 eV pulses, then only intralayer K_W_-K_W_ A1s excitons are optically excited and decay in a cascaded transition via layer hybridized K_W_-Σ excitons to interlayer K_W_-K_Mo_ excitons, as we have discussed in detail in our earlier work (*8*, *27*) (i.e., K_W_-K_W_ → K_W_-Σ → K_W_-K_Mo_; Fig. 2A, left-hand side). In the single-particle picture, this cascaded transition can be associated with the transfer of the exciton’s electron across the TMD interface (Fig. 1B).

Complementary, if the hole-transfer process across the WSe_2_/MoS_2_ interface is considered (Fig. 1C), then the dynamics must be initiated by an excitation of MoS_2_ A1s excitons with 1.9 eV light pulses (K_Mo_-K_Mo_ excitons in Fig. 2A, right-hand side). Exploiting the density matrix formalism, we calculate the excitonic energy landscape (details below), and track the exciton dynamics, finding that the most efficient mechanism to form interlayer K_W_-K_Mo_ excitons occurs via layer hybridized Γ-K_Mo_ excitons, where the exciton’s electron resides in the K_Mo_ valley of MoS_2_ and the exciton’s hole can be found in the layer-hybridized valence bands at the Γ valley (*38*). Hence, the hole-transfer dominantly occurs via the K_Mo_-K_Mo_ → Γ-K_Mo_ → K_W_-K_Mo_ exciton cascade.

To differentiate the spectral contributions of different excitons in the experiment, we apply our setup for femtosecond momentum microscopy (*39*, *40*) that provides direct access to the photoemission energy-momentum fingerprint of excitons (Fig. 2, B to E). In Fig. 2E, the momentum map of the intralayer K_Mo_-K_Mo_ exciton is shown after resonant optical excitation with 1.9-eV pump pulses. Photoelectrons are detected at the in-plane momenta of the K_Mo_ and K′_Mo_ valleys (0 ps). For better visibility, the Brillouin zone of MoS_2_ is overlaid in dark red. Because 1.9-eV pump photons also non-resonantly excite K_W_-K_W_ excitons in WSe_2_, the momentum map in Fig. 2C shows photoemission yield at the K_W_ and K′_W_ valleys of WSe_2_ (orange hexagon, 0 ps). Note that the Brillouin zone of WSe_2_ is rotated by 9.8^∘^ ± 0.8^∘^ with respect to MoS_2_. Moreover, weak photoemission yield from hybrid K_W_-Σ excitons is detected at the Σ and Σ' valleys (grey hexagon). At a pump-probe delay of 10 ps (Fig. 2D), the major part of the intralayer excitons has decayed either via the electron- or the hole-transfer process, and spectral yield is dominated by the energetically most stable excitation, i.e., the interlayer K_W_-K_Mo_ excitons (fig. S4). For these interlayer excitons, the electron and the hole contributions are now separated between both monolayers of the heterostructure, and the exciton photoemission momentum fingerprint has to be described within the moiré mini-Brillouin zones built up by the κ valleys whose in-plane momentum can be constructed by the reciprocal lattice vectors of WSe_2_ and MoS_2_ (Fig. 2D, black hexagon) (*8*, *26*).

### Hole- and electron-transfer dynamics

**Fig. 3.**
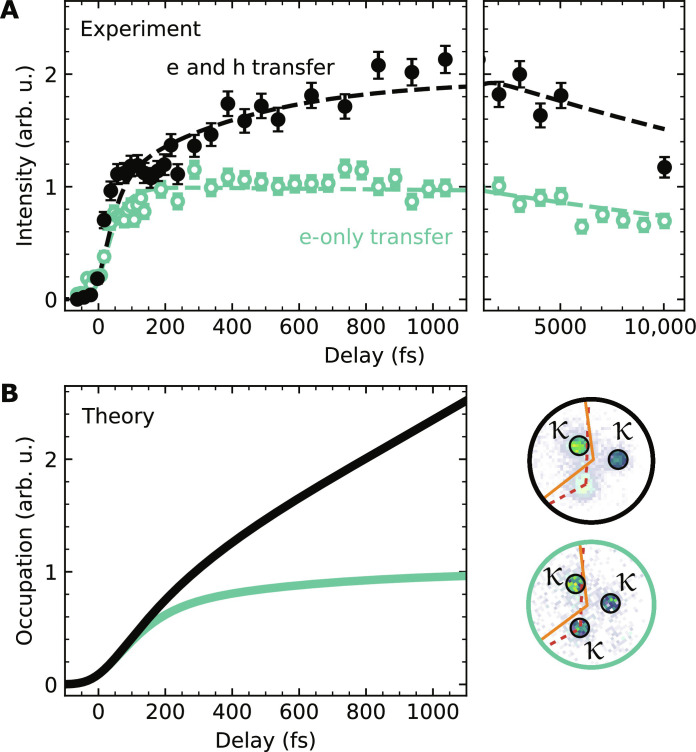
Femtosecond-to-picosecond evolution of the hole- and electron-transfer dynamics. (**A**) Direct comparison of the interlayer K_W_-K_Mo_ exciton formation dynamics if the heterostructure is excited resonantly to the intralayer K_W_-K_W_ exciton energy of WSe_2_ (1.7 eV, green circles) or the intralayer K_Mo_-K_Mo_ exciton of MoS_2_ (1.9 eV, black circles). While the electron-only transfer process (1.7 eV) leads to a saturation of photoemission yield from interlayer K_W_-K_Mo_ excitons on the <200-fs timescale, the combined electron- and hole-transfer dynamics (1.9 eV) leads to an increasing photoemission yield up to 1 ps. The momentum-filtered regions of interest (black circles) used in the 1.7-eV (green contour) and 1.9-eV (black contour) measurements are shown in the bottom panel. The κ valley that overlaps with the original K_Mo_ valley is excluded in the analysis of the 1.9-eV measurement. (**B**) Microscopic model calculations of the interlayer K_W_-K_Mo_ exciton formation dynamics. The green curve describes the temporal evolution of the occupation of interlayer K_W_-K_Mo_ excitons after photoexcitation of intralayer K_W_-K_W_ excitons. For the black curve, the interlayer K_W_-K_Mo_ exciton formation dynamics is induced by the initial excitation of intralayer K_W_-K_W_ and K_Mo_-K_Mo_ excitons. Note that the model calculations do not include additional decay processes.

Having identified the exciton fingerprints in the photoemission experiment, we can now proceed with the analysis of the hole-transfer dynamics. For this, fig. S4 provides an overview of the pump-probe delay-dependent evolution of photoemission intensity from intralayer K_Mo_-K_Mo_ and K_W_-K_W_ excitons, the hybrid K_W_-Σ exciton, and the interlayer K_W_-K_Mo_ exciton after optical excitation with 1.9 eV (fluence: 140 μJ/cm^2^; optically excited exciton densities of 7 ×10^11^ and 3.5 ×10^12^ cm^−2^ in WSe_2_ and MoS_2_ (*41*), respectively). The formation and thermalization dynamics of all accessible excitons indicate that electron- and hole-transfer processes contribute to the formation of interlayer K_W_-K_Mo_ excitons, which, in consequence, we have to distinguish. To do so, we directly compare the interlayer K_W_-K_Mo_ exciton rise time for 1.7- and 1.9-eV pumping. In Fig. 3A, the black data points show the pump-probe delay-dependent buildup of interlayer K_W_-K_Mo_ exciton photoemission intensity that is formed by electron- and hole-transfer processes (1.9-eV pump photons). For comparison, the green data points show the pump-probe delay-dependent buildup of the interlayer K_W_-K_Mo_ exciton intensity that is formed only via the electron transfer process (1.7-eV pump photons, fluence: 280 μJ/cm^2^, exciton density: 5.4 ×10^12^ cm^−2^). It is directly obvious that there is a strong hierarchy of timescales for the electron- and hole-transfer processes: When considering the electron-only transfer process (green symbols), the interlayer exciton signal increases rapidly with pump-probe delay and saturates on the sub-200-fs timescale. A quantitative evaluation with rate equation modeling yields a formation time of *t*_e − transfer_ = 40 ± 10 fs (see Supplementary Text). In contrast, the joint buildup of interlayer K_W_-K_Mo_ excitons via electron- and hole-transfer processes after 1.9-eV excitation saturates on the 1-ps timescale (black symbols). For further analyzing this dataset, we assume that the 1.9-eV pump pulses excite A1s excitons in WSe_2_ and MoS_2_ in a 1:5 ratio, as estimated from the optical absorption coefficient of both monolayers (*41*), and take the already deduced electron-transfer time *t*_e − transfer_ = 40 ± 10 fs into account. From this fit, we extract *t*_h − transfer_ = 2.2 ± 1 ps, which is more than an order of magnitude larger than the electron-transfer time *t*_e − transfer_ (see rate equation analysis based on fig. S3).

Hence, our experimental data imply that the interlayer hole-transfer mechanism across the WSe_2_/MoS_2_ heterointerface is substantially slower compared to the electron-transfer mechanism. To understand our findings on a microscopic footing, we exploit the density matrix formalism to derive excitonic equations of motion within the energy landscape of excitons shown in Fig. 2A and fig. S7 (see details in Supplementary Text) (*38*, *42*). Here, we incorporate exciton-light and exciton-phonon interaction and assume again that the 1.9-eV pump pulses excite A1s excitons in WSe_2_ and MoS_2_ in a 1:5 ratio (*41*). We find an excellent qualitative agreement of the microscopic model calculations (Fig. 3B) with the experimentally quantified rise time (Fig. 3A) of interlayer K_W_-K_Mo_ excitons: The electron-only transfer process saturates for delays <200 fs (green), while the combined electron- and hole-transfer dynamics lead to an increasing interlayer K_W_-K_Mo_ exciton occupation for substantially longer delays (black). Hence, in experiment and theory, we find that the electron-transfer dynamics is roughly one order of magnitude faster than the hole-transfer dynamics.

To understand this drastic difference in the rise time of interlayer K_W_-K_Mo_ exciton formation via the electron- versus the hole-transfer process, we evaluate the calculated exciton dynamics in more detail and make two major observations: First, it is important to realize that the exciton energy difference between the optically excited intralayer exciton and the interlayer exciton is roughly 200 meV larger in the case of 1.9-eV excitation, which initiates the hole-transfer process (see exciton energies in Fig. 2A and fig. S7). The dissipation of this extra amount of energy via exciton-phonon scattering events with typical phonon frequencies of 0.03 eV (*43*) leads to overall slower hole-transfer dynamics (arrows in Fig. 2A) (*42*, *44*). In addition, the first step of the exciton cascade leading to the formation of either K_W_-Σ or Γ-K_Mo_ excitons in the electron- and hole-transfer process, respectively, is markedly different. In the first Brillouin zone, the Σ and Σ' valleys are each threefold degenerate, while there is only one Γ valley (Fig. 2A, inset). Therefore, the density of final states for the K_W_-K_W_ → K_W_-Σ versus the K_Mo_-K_Mo_ → Γ-K_Mo_ transition is notably different (*42*, *43*, *45*–*47*). In consequence, hybrid K_W_-Σ excitons are more efficiently formed than hybrid Γ-K_Mo_ excitons, favoring faster interlayer exciton formation dynamics for the electron-transfer channel compared to the hole-transfer channel.

Last, we want to point out two important deviations in the exciton dynamics between experiment and theory. First, on the few picosecond timescale, we find that the calculated occupation of interlayer K_W_-K_Mo_ excitons increases up to ≈4 ps and is composed of a 1:5 ratio of interlayer excitons that are formed from A1s excitons initially excited in the WSe_2_ and MoS_2_ layers (fig. S8). In contrast, in the experiment, the respective photoemission intensity saturates at roughly 1 ps and the 1:5 ratio cannot be identified (1.9-eV excitation; Fig. 3). This deviation between experiment and theory can be understood by the fact that radiative and defect-assisted decay processes of intralayer, hybrid, and interlayer excitons with lifetimes ranging from 1 ps to tenths of picoseconds (*8*, *27*, *34*, *35*) are not included in the model calculations. Hence, the model calculations overestimate the exciton occupation at large pump-probe delays.

Second, we find that the experimental data for 1.7- and 1.9-eV excitation rises faster than estimated from the model calculations (sub-200-fs timescale in Fig. 3). This deviation could be related to the fact that the model calculations do not consider exciton-exciton scattering events, which might already contribute to the dynamics in the experiment (*25*, *48*, *49*). Although an in-depth pump fluence-dependent analysis of these dynamics appears to be highly interesting, it is beyond the scope of this manuscript, and, in the following, we focus on the identification of a spectroscopic fingerprint of the hole-transfer process.

### The spectroscopic signature of a correlated hole-transfer process

On the basis of this hierarchy of timescales between the electron- and the hole-transfer process, it is possible to separate the interlayer exciton formation dynamics: For delays >200 fs, the change in the exciton photoemission yield from the interlayer K_W_-K_Mo_ exciton is mainly caused by hole-transfer processes. Hence, the final ambition of our work is the unambiguous discrimination of the photoemission spectral signature of intralayer K_Mo_-K_Mo_ and interlayer K_W_-K_Mo_ excitons, where, in both cases, the electron contribution to the exciton is situated in the conduction band minimum of the MoS_2_ layer (compare Fig. 1C).

**Fig. 4.**
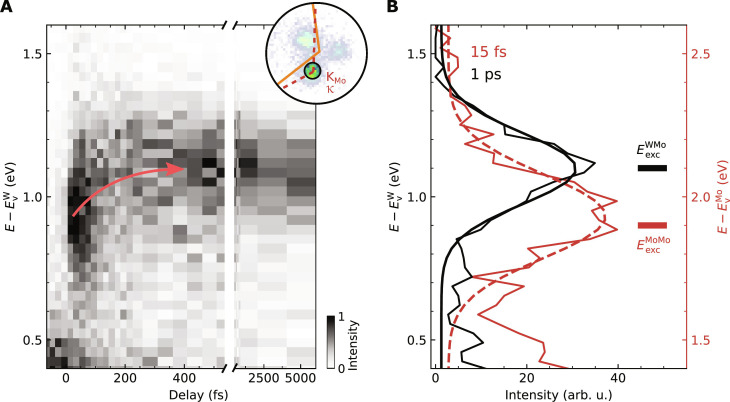
Coulomb correlation–induced excitonic energy fingerprints. (**A**) Pump-probe delay evolution of the energy distribution curves (EDCs) filtered at the momentum region of the K_Mo_ valley of MoS_2_ (region of interest indicated in the inset, 1.9-eV excitation). At this high-symmetry point, photoemission yield from intralayer K_Mo_-K_Mo_ and interlayer K_W_-K_Mo_ excitons is expected (see Fig. 1C). As intralayer K_Mo_-K_Mo_ excitons decay and form interlayer K_W_-K_Mo_ excitons, the peak maxima of the photoelectron energy shows an upshift by $\Delta E_{\text{PES}}^{h-\text{transfer}}=0.17\pm 0.04\;\text{eV}$ (curved arrow). (**B**) Selected EDCs for pump-probe delays of 15 fs (dark red) and 1 ps (black) illustrating an energetic upshift of the exciton photoemission signal. The horizontal bars indicate expected photoelectron energies for the intralayer K_Mo_-K_Mo_ (dark red) and interlayer K_W_-K_Mo_ (black) excitons calculated with Eq. 1 and data from photoluminescence measurements (*56*, *57*). The left and right energy axes in black and dark red show the corresponding energy scales with respect to the valence band maximum of WSe_2_ and MoS_2_.

In the most naive picture of photoemission, it might be expected that trARPES only yields information on the exciton’s electron. Hence, the experiment would not distinguish between photoelectrons being emitted from the conduction band minimum of MoS_2_, irrespective of whether they result from the breakup of intralayer K_Mo_-K_Mo_ or interlayer K_W_-K_Mo_ excitons (Fig. 1C). However, it is known that the spectral function in photoemission contains information about many-body interactions (*50*), and this is also the case for the correlated electron-hole pair. This leads to a very nonintuitive and intriguing experimental observation. Figure 4A shows the pump-probe delay evolution of energy distribution curves (EDCs) filtered for photoelectron yield at the κ valley, whose momentum coincides with the K_Mo_ valley, i.e., the momentum region where photoelectron yield from intralayer K_Mo_-K_Mo_ and interlayer K_W_-K_Mo_ excitons is expected (Fig. 4A, inset). Astonishingly, we find that the energy of the photoelectrons shifts up as a function of pump-probe delay from $E-E_{v}^{W}=0.93\pm 0.03\;\text{eV}$ at 15 fs to $E-E_{v}^{W}=1.10\pm 0.03\;\text{eV}$ at 1 ps, i.e., a shift of $\Delta E_{\text{PES}}^{h-\text{transfer}}=0.17\pm 0.04\;\text{eV}$ (Fig. 4B). At first glance, this is an unexpected observation: In temporal overlap of the pump and the probe laser pulses, the optical excitation deposits energy into the system, and the system subsequently relaxes from its excited state to energetically more favorable states via scattering processes. In consequence, energy-resolved pump-probe photoemission spectroscopies of single-particle charge carriers typically show that the mean kinetic energy of the photoelectrons decreases with pump-probe delay (*51*). An increasing mean kinetic energy might indicate higher-order scattering processes such as Auger recombination (*49*, *52*). For Auger recombination, however, we would expect to observe a decreasing mean kinetic energy on the few-picosecond timescale as the overall exciton density and thus the efficiency for Auger recombination decreases. However, the long-time evaluation of the mean photoelectron energy clearly excludes this scenario (Fig. 4A). In addition, by evaluating the pump-probe delay evolution of the energy position of the MoS_2_ valence band maxima, we can exclude a photoinduced renormalization of the band energies (*53*, *54*) (fig. S5). We thus search for the origin of the apparent increase of the mean kinetic energy beyond the single-particle picture, i.e., in the photoemission from excitons whose occupation is dynamically transferring from intralayer K_Mo_-K_Mo_ to interlayer K_W_-K_Mo_ excitons.

So far, we have referenced the energies of all emitted single-particle photoelectrons to the valence band maximum of WSe_2_ (left energy axis in Fig. 4B). However, especially for the intralayer K_Mo_-K_Mo_ exciton that fully resides in the MoS_2_ layer, this is clearly not the intrinsically relevant energy axis. We overcome this shortcoming by using an energy scale that is more direct to photoemission from excitons by relating the total energy before (*E* = *E*_0_ + *E*_exc_ + *ℏ*ω) and after (*E* = *E*_0_ − *E*_hole_ + *E*_elec_) the breakup of the correlated electron-hole pair (*55*). Here, *E*_exc_ is the energy necessary to resonantly excite an exciton with a two-particle binding energy *E*_bin_ (compare exciton energy landscape in Fig. 2A); *E*_hole_ and *E*_elec_ denote the energy of the single-particle hole and electron state after the breakup of the exciton, respectively; *E*_0_ is the ground state energy and *ℏ*ω is the photon energy. As energy needs to be conserved when the exciton is broken, the energy of the detected single-particle electron can be expressed as

$$E_{\text{elec}}=E_{\text{hole}}+E_{\text{exc}}+\hbar \omega$$ (1)

Therefore, Eq. 1 fixes the energy of the single-particle hole *E*_hole_ remaining in the sample as the natural reference point of the photoelectron energy axis for each probed exciton (at a given probe photon energy *ℏ*ω). For the intralayer K_Mo_-K_Mo_ excitons and the interlayer K_W_-K_Mo_ excitons, respectively, the valence band maxima of MoS_2_ ($E_{v}^{\text{Mo}}$) and WSe_2_ ($E_{v}^{W}$) set the energy scale [see Fig. 1 (B and C) and band energies labeled in Fig. 2B)]. Following Eq. 1, we can directly quantify the exciton energies of intralayer K_Mo_-K_Mo_ and interlayer K_W_-K_Mo_ excitons from the photoemission data to $E_{\text{exc}}^{\text{MoMo}}=1.93\pm 0.08\;\text{eV}$ and $E_{\text{exc}}^{\text{WMo}}=1.10\pm 0.03\;\text{eV}$, respectively, which are in excellent agreement with earlier results obtained with photoluminescence spectroscopy [($E_{\text{exc},\text{PL}}^{\text{MoMo}}=1.9\;\text{eV}$ and $E_{\text{exc},\text{PL}}^{\text{WMo}}=1.1\;\text{eV}$; horizontal lines in Fig. 4B)] (*56*, *57*). In consequence, we can explain the experimentally observed upshift of the photoelectron energy by $\Delta E_{\text{PES}}^{h-\text{transfer}}=0.17\pm 0.04\;\text{eV}$ with the energy difference between the single-particle electron final states *E*_elec_ of the interlayer K_W_-K_Mo_ and the intralayer K_Mo_-K_Mo_ excitons, i.e., with $\left(E_{v}^{W}+E_{\text{exc}}^{\text{WMo}}+\hbar \omega \right)-\left(E_{v}^{\text{Mo}}+E_{\text{exc}}^{\text{MoMo}}+\hbar \omega \right)\approx 0.17\;\text{eV}$ (with $E_{v}^{W}-E_{v}^{\text{Mo}}=1.00\pm 0.07\;\text{eV}$, see Fig. 2B). Hence, the energetic upshift is a direct consequence of the breakup of the correlated electron-hole pair during the photoemission process.

Although the photoelectron energy increases during the hole-transfer process, we strongly emphasize that the overall energy of the system relaxes by $\Delta E_{\text{exc}}^{h-\text{transfer}}=E_{\text{exc}}^{\text{WMo}}-E_{\text{exc}}^{\text{MoMo}}=-0.83\pm 0.09\;\text{eV}$ (see Fig. 2A). Consistently, if the same analysis is performed for the electron-only transfer process after photoexcitation with 1.7 eV pump pulses, then we find a reduction of the overall exciton energy by $\Delta E_{\text{exc}}^{e-\text{transfer}}=E_{\text{exc}}^{\text{WMo}}-E_{\text{exc}}^{\text{WW}}=-0.46\pm 0.07\;\text{eV}$ (fig. S6). In this case, where the exciton’s hole remains in the WSe_2_ VBM (Fig. 1B), the reduction of the exciton energy directly translates to a reduction of the single-particle photoelectron energy ($\Delta E_{\text{PES}}^{e-\text{transfer}}=-0.46\pm 0.07\;\text{eV}$). Therefore, as expected, interlayer charge transfer always leads to a reduction of the exciton energy *E*_exc_, which might, however, result in an up- or a downshift of the photoelectron energy in the photoemission spectrum.

## DISCUSSION

We have shown that femtosecond momentum microscopy is a powerful tool to study the correlated interaction between the exciton’s electron and hole in twisted semiconductor heterostructures. Exemplarily, we show that the photoelectron of the correlated two-particle exciton contains direct information about the hole state. We use this correlation in combination with microscopic and material-specific theory to directly follow an ultrafast interlayer hole-transfer process that would otherwise be elusive. Our work opens up means for the future study of correlated states of matter in two-dimensional quantum materials.

## MATERIALS AND METHODS

The time- and angle-resolved photoemission data are measured with a time-of-flight momentum microscope (Surface Concept) (*58*, *59*) that is connected to a table-top high harmonic generation beamline driven by a 300-W fiber laser system (AFS Jena) (*40*, *60*). The overall experimental setup and its application to exfoliated two-dimensional materials are described in (*39*) and (*8*), respectively.

In all experiments, the exciton dynamics are induced by resonant optical excitation of the A1s-excitons of WSe_2_ or MoS_2_. Therefore, 1.7- and 1.9-eV pump pulses with a duration of 50 fs are used (*s*-polarized), respectively. After a variable pump-probe delay, photoemission is induced by 26.5-eV light pulses (20 fs, *p*-polarized).

For the characterization of the temporal resolution and the determination of absolute time zero of the experiment, we have measured the pump-probe delay-dependent photoemission yield of sidebands of the valence bands formed due to the laser-assisted photoelectric effect (*40*, *61*). In fig. S1, a cross-correlation of the pump and probe laser pulse is shown, where both laser pulses are *p*-polarized (1.9-eV pump pulses). The gray line is a Gaussian fit to the data yielding a full width at half maximum of 60 ± 5 fs.

The 9.8^∘^ ± 0.8^∘^ twisted WSe_2_/MoS_2_ heterostructure is stamped onto a 20- to 30-nm-thick hBN (*62*) spacer layer and a p ^+^-doped native oxide silicon waver. Before the momentum microscopy experiments, the sample is annealed for 1 hour to 670 K. Details on the sample fabrication and characterization (e.g., twist angle) are described in (*8*).

## Supplementary Materials

### This PDF file includes:

Supplementary Text Figs. S1 to S8 Table S1 References
