# Supplementary material for: Probing electron-hole Coulomb correlations in the exciton landscape of a twisted semiconductor heterostructure
Source: Sci Adv. 2024 Feb 7;10(6):eadi1323. doi: 10.1126/sciadv.adi1323 (PMC10849592; doi:10.1126/sciadv.adi1323)
Supplement: Supplementary file 1 — Supplementary Text Figs. S1 to S8 Table S1 References [file sciadv.adi1323_sm.pdf]

Science Advances
AAAS

Supplementary Materials for

Probing electron-hole Coulomb correlations in the exciton landscape of a twisted semiconductor heterostructure

Jan Philipp Bange *et al.*

Corresponding author: Marcel Reutzel, marcel.reutzel@phys.uni-goettingen.de;
Stefan Mathias, smathias@uni-goettingen.de

Sci. Adv. **10**, eadi1323 (2024)
DOI: 10.1126/sciadv.adi1323

This PDF file includes:

Supplementary Text
Figs. S1 to S8
Table S1
References

Supplementary Text

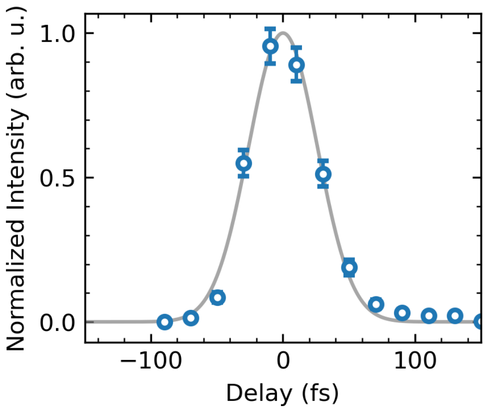

Figure S1: **Cross-correlation measurement of the pump and probe laser pulses as obtained in the photoemission experiment due to the measurement of photoemission sidebands formed due to the laser-assisted photoelectric effect.**

Correction of rigid band shifts

As a result of pump- and probe-induced space-charge and surface photovoltage effects, we observe transient energy shifts of the momentum-integrated photoemission spectrum by ±80 meV (Fig. S2) (*63*) . This energy offset is extracted from the data by fitting the spectral weight maxima at ≈-2.4 eV (Fig. S2B) for each pump-probe delay. The blue and the black data points show the pump-probe dependence of this peak before and after correction, respectively. The correction is done prior to the data analysis discussed in the main text.

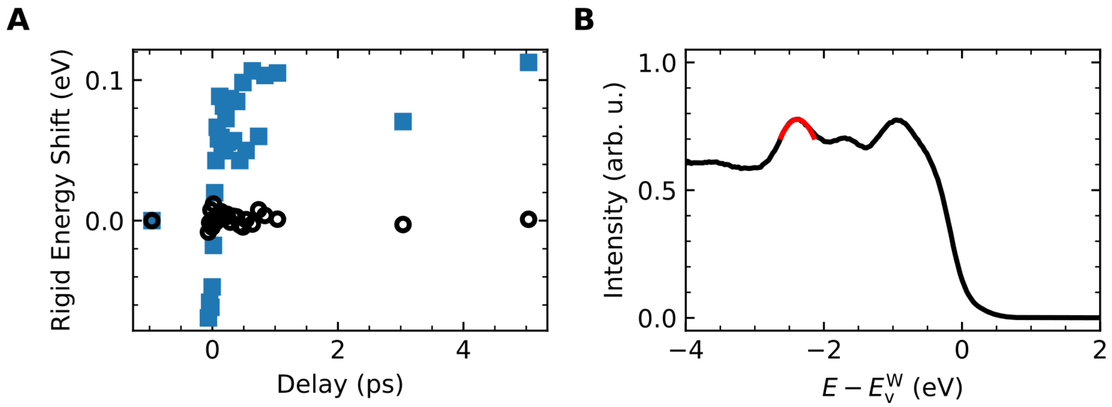

Figure S2: **Correction of rigid energy shifts.** (**A**) At each pump-probe delay, the momentum-integrated energy spectrum has a distinct rigid energy shift (blue squares) that is evaluated by fitting the red marked peak close to E-E_v^W= -2.4 eV in (**B**). After the correction of this rigid energy shift, all energy-distribution-curves are aligned (black circle data points in (**A**)).

Quantitative analysis of the exciton dynamics - Rate equation modelling

Fig. 3A in the main text and Fig. S3C show the pump-probe delay-dependent photoemission intensity from interlayer K_W-K_{Mo} excitons in the case that electron- and hole-transfer processes (black circles, 1.9 eV) or that electron-only-transfer processes (green circles, 1.7 eV) contribute. In order to quantitatively analyze the characteristic formation dynamics, we apply a rate equation model to fit the experimental data. The model is schematically shown in Fig. S3A and S3B and the rate equations are listed in the following

$$\frac{dN_{\mathrm{W}}}{dt} = g_{\mathrm{W}}(t) - \frac{N_{\mathrm{W}}}{t_{\mathrm{e-transfer}}}, \tag{S1}$$

$$\frac{dN^{\mathrm{e-only}}_{\mathrm{K_W-K_{Mo}}}}{dt} = \frac{N_{\mathrm{W}}}{t_{\mathrm{e-transfer}}} - \frac{N^{\mathrm{e-only}}_{\mathrm{K_W-K_{Mo}}}}{\tau_{\mathrm{decay}}}, \tag{S2}$$

$$\frac{dN_{\mathrm{Mo}}}{dt} = g_{\mathrm{Mo}}(t) - \frac{N_{\mathrm{Mo}}}{t_{\mathrm{h-transfer}}} - \frac{N_{\mathrm{Mo}}}{\tau_{\mathrm{intra}}}, \tag{S3}$$

$$\frac{dN^{\mathrm{e\&h}}_{\mathrm{K_W-K_{Mo}}}}{dt} = \frac{N_{\mathrm{Mo}}}{t_{\mathrm{h-transfer}}} + \frac{N_{\mathrm{W}}}{t_{\mathrm{e-transfer}}} - \frac{N^{\mathrm{e\&h}}_{\mathrm{K_W-K_{Mo}}}}{\tau_{\mathrm{decay}}}. \tag{S4}$$

N_{W} and N_{Mo} are the intralayer exciton occupation in the WSe_2 and MoS_2 layer, respectively, that are populated with Gaussian shaped excitation $g_{\mathrm{W}}(t)$ and $g_{\mathrm{Mo}}(t)$ $(\mathrm{FWHM} = 50$ fs). As the probe laser pulse duration is sufficiently short (20 fs), it is neglected in the rate equation fit. $t_{\mathrm{e-transfer}}$ and $t_{\mathrm{h-transfer}}$, respectively, describe the electron- and hole-transfer times from the initial N_{W} and N_{Mo} states into the final $N^{\mathrm{e\&h}}_{\mathrm{K_W-K_{Mo}}}$ and $N^{\mathrm{e-only}}_{\mathrm{K_W-K_{Mo}}}$ states. Based on the absorption coefficients of MoS_2 and WSe_2 at excitation with 1.9 eV laser pulses (*41*), we expect to excite the respective intralayer A1s excitons in a 1:5 ratio, i.e., $g_{\mathrm{Mo}}(t) = 5 \cdot g_{\mathrm{W}}(t)$. It is important to note that because of photoemission matrix element effects (*25*), the direct correlation of the photoemission intensity from excitons to the excitonic occupation is not possible. Hence, the excitation ratio cannot experimentally be extracted from the pump-probe delay-dependent analysis of the photoemission intensity from intralayer K_W-K_W and K_{Mo}-K_{Mo} excitons (Fig. S4).

At first glance, it might be expected the the electron-transfer and hole-transfer processes also contribute to the interlayer exciton occupation with a ratio of 1:5. However, this assumption neglects additional decay processes that must be taken into account. In particular, we observe that the hole-mediated interlayer K_W-K_{Mo} exciton photoemission yield increases on an time-scale of up to 1 ps (Fig. 3A). On this comparably long time-scale, radiative and defect-assisted decay processes of intralayer and hybrid excitons with lifetimes in the regime of a few picoseconds clearly become relevant (*8, 27, 34, 35*). Hence, not all initially excited intralayer excitons are converted to interlayer excitons. In order to capture these processes in our fit routine, we add the decay constant τ_{intra} as a free fit parameter to equation (S3). Moreover, the interlayer exciton state depopulates with a fixed decay time of $\tau_{\text{decay}} = 33$ ps, which was estimated from an exponential decay fit to the long-term dynamics (*8*).

In our analysis, we first fit the 1.7 eV pumped data set. In the fit routine, equations (S1) and (S2) are solved numerically yielding the delay-dependent evolution of the state $N^{\text{e-only}}_{\text{K}_\text{W}-\text{K}_\text{Mo}}$. This is then compared to the data. Fit parameters are amplitude of the excitation and electron transfer time $t_{\text{e-transfer}}$. Optimization for best fit parameters yields $t_{\text{e-transfer}} = 40 \pm 10$ fs.

In a second step, we fit the 1.9 eV data. Equations (S1), (S3) and (S4) are solved by incorporating the fixed electron-only transfer time $t_{\text{e-transfer}} = 40 \pm 10$ fs from the previous fit and $g_{\text{Mo}}(t) = 5 \cdot g_{\text{W}}(t)$ (see above). The remaining fit parameters are the amplitude of the excitation, the hole-transfer time $t_{\text{h-transfer}}$ and the decay time τ_{intra} of the initial N_{Mo} state. The optimization for the best fit parameters yields a hole-transfer time of $t_{\text{h-transfer}} = 2.2 \pm 1$ ps. Moreover, the fitted decay time $\tau_{\text{intra}} = 600 \pm 200$ fs is in a reasonable order in comparison to earlier work on radiative and defect decay processes of intralayer and hybrid excitons (*8, 27, 34, 35*).

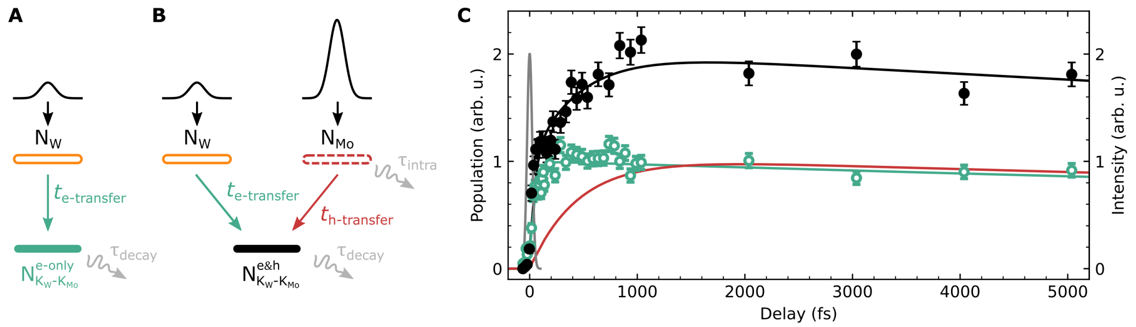

Figure S3: **Quantitative analysis of the interlayer K_W-K_{Mo} exciton formation dynamics.** (**A**) Schematic overview of the rate equation model: Exciton population in the WSe_2 layer N_W is excited by a Gaussian pump pulse. Electron charge-transfer leads to the formation of interlayer K_W-K_{Mo} excitons ($N^{e-only}_{K_W-K_{Mo}}$) with the scattering time $t_{e-transfer}$. (**B**) Exciton population in the MoS_2 (WSe_2) layer N_{Mo} (N_W) is excited by a Gaussian pump pulse. Hole (electron) charge-transfer leads to the formation of interlayer K_W-K_{Mo} excitons ($N^{e\&h}_{K_W-K_{Mo}}$) with the scattering time $t_{h-transfer}$ ($t_{e-transfer}$). (**C**) The pump-probe delay-dependent build-up of photoemission intensity of interlayer K_W-K_{Mo} excitons is shown after resonant excitation of K_{Mo}-K_{Mo} excitons in MoS_2 (1.9 eV, black circles) and after resonant excitation of K_W-K_W excitons in WSe_2 (1.7 eV, green circles), respectively. Note that resonant excitation of K_{Mo}-K_{Mo} excitons also leads to off-resonant excitation of K_W-K_W excitons, so that the interlayer exciton occupation is build-up both by hole and electron transfer. Solid lines depict best fit results of the rate equation model. Green and dark red solid lines describe the proportion of the interlayer exciton population that is created due to the electron-only ($N^{e-only}_{K_W-K_{Mo}}$) and hole-only charge transfer, respectively. The black line corresponds to the sum of electron- and hole-transfer processes $N^{e\&h}_{K_W-K_{Mo}}$.

Femtosecond dynamics of intra- and interlayer excitons

Figure S4 shows an overview of the pump-probe delay-dependent evolution of photoemission intensity for all measured excitons after excitation with 1.9 eV light pulses. The first two rows show the optical excitation of intralayer K_W-K_W excitons (orange) and the subsequent formation of hybrid K_W-Σ excitons (grey). The bottom two panels show the photoemission intensity of selected κ valleys of the moiré mBz. In the case that the κ valley overlaps with the K_{Mo} valley (dark red), photoemission intensity is composed of signal from intralayer K_{Mo}-K_{Mo} and interlayer K_{Mo}-K_W excitons. This κ valley is evaluated in Fig. 4 of the main text. Complementary, if the two κ valleys are evaluated (black) that do not overlap with the K_{Mo} valley, only photoemission signal from interlayer K_{Mo}-K_W excitons is detected. These data are shown in Fig. 3A of the main text. Error bars of the data depicted in Fig 3A of the main text and Figure S1, S3B and S4 show the 1σ interval of the Poisson distributed count statistics.

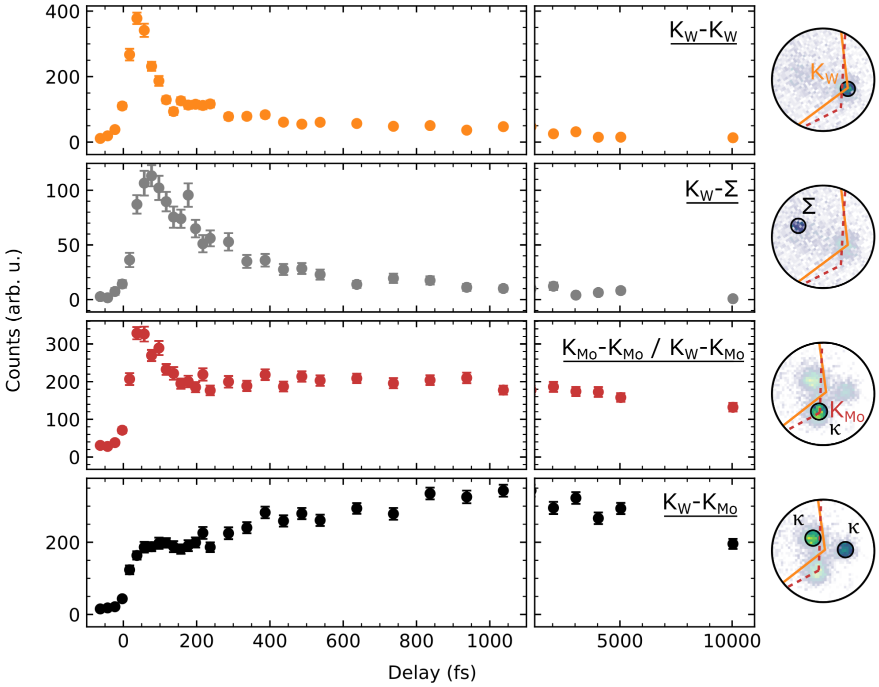

Figure S4: **Femtosecond intra- to interlayer exciton transfer dynamics.** The dynamics for the different exciton signals are depicted: K_W-K_W (orange), K_W-Σ (grey), K_{Mo}-K_{Mo} and K_W-K_{Mo} (dark red) and K_W-K_{Mo} (black). The round insets show the position of the momentum apertures used for filtering the exciton photoemission signatures. If the κ valley coincides with the K_{Mo} valley (dark red), photoemission yield is composed of contributions from intralayer K_{Mo}-K_{Mo} and interlayer K_W-K_W excitons (see Fig 4 of the main text). In the case that those high-symmetry points do not overlap (black), only photoemission signal from interlayer K_W-K_{Mo} excitons is detected (see Fig. 3 of the main text). Momentum filters have a diameter of 0.17 Å^{-1} and the signal is summed over all 6 corners of the first Brillouin zone. Energy binning is 2.3-1.3 eV for K_W-K_W and K_W-Σ and 1.42-0.75 eV for K_W-K_{Mo} and K_{Mo}-K_{Mo}.

Excluding photoinduced band renormalizations

The major spectroscopic signature of interest in our manuscript is a pump-probe delay-dependent upshift of the energy of photoelectrons being emitted from excitons (Fig. 4). We attribute this energy upshift to the formation of interlayer K_{Mo}-K_W excitons from intralayer K_{Mo}-K_{Mo} excitons. However, it is well-known that photo-induced band renormalizations (*53*) can lead to a similar shift of photoemission signatures (*54*), which, hence, must be excluded.

In addition to photoemission signals from excitons, the multidimensional data acquisition scheme allows to monitor the energetic position of the occupied valence band of MoS_2. If the electronic bands would renormalize in response to the optical excitation, we would expect to observe an energetic shift of this occupied valence band (*54*). In Fig. S5, we directly compare the energy position of the MoS_2 valence band and the excitonic photoemission signal. We observe that after the excitation with the pump pulse the excitonic peak position at the K_{Mo} point exhibits an upshift, while the valence band maximum of the MoS_2 layer remains comparably constant. Hence, we can exclude photo-induced band renormalizations as the origin for the energetic upshift of the main photoemission signal in Fig. 4.

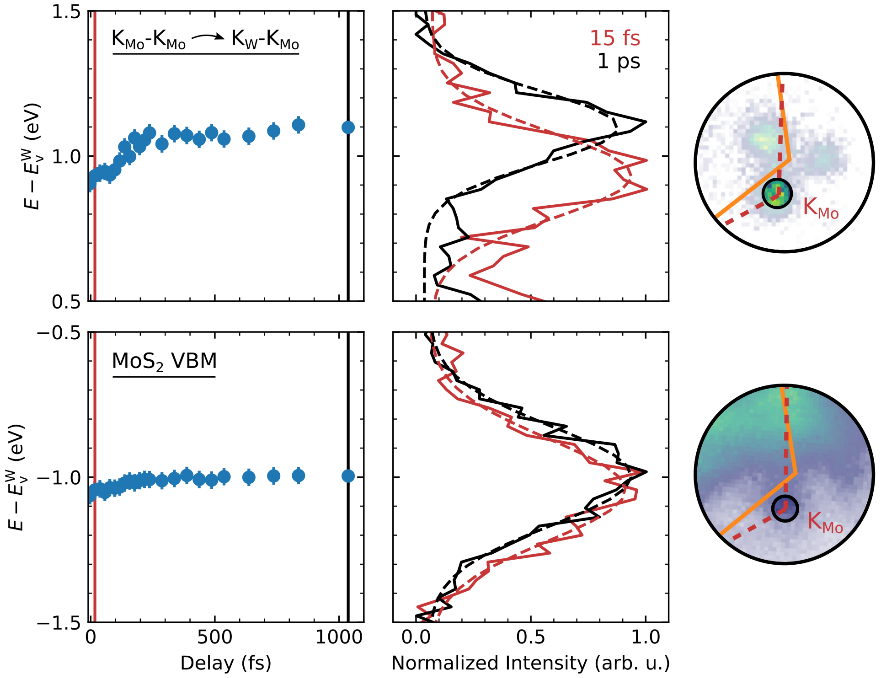

Figure S5: **Excluding photo-induced band renormalizations.** The top and the bottom rows show the peak position of the exciton photoemission signal and the MoS_2 valence band maximum, respectively. In the middle panels, selected EDCs taken at the K_{Mo} valley are shown for 15 fs (dark red) and 1 ps (black). In the left panels, the fitted peak maxima of such EDCs are plotted as a function of pump-probe delay. The right panels show the filtered momentum regions (black circles), whereas the momentum-momentum maps are taken at the respective energies of the excitonic photoemission signal and the MoS_2 valence band maximum.

Exciton energy relaxation during electron- and hole-transfer process

In Fig. 4 of the main text we show an upshift of the photoelectron energy of the exciton signal caused by the hole charge-transfer process. Figure S6 depicts this photoemission signature when pumping with 1.9 eV (Fig. S6A) in comparison to the photoemission signature upon 1.7 eV pumping (Fig. S6B). For the latter case that initiates the electron charge-transfer process, we observe a reduction in the photoelectron energy in agreement with our earlier report (*8*). We note that in both cases the exciton relaxes towards the energetically lowest lying state in the overall exciton energy landscape, i.e. to the interlayer exciton, as shown by the analysis in the main text.

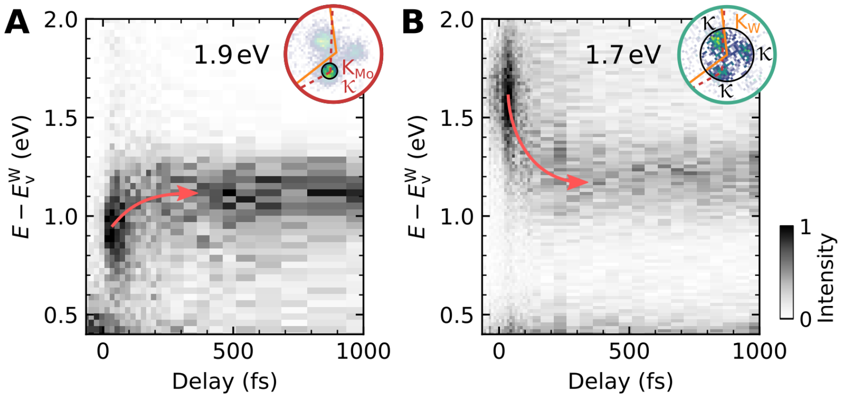

Figure S6: **Pump-probe delay evolution of the exciton energy fingerprints for the case of the hole- and the electron-transfer process.** (**A**) Pump-probe delay evolution of the momentum filterd energy-distribution curves (EDC) with a pump energy of 1.9 eV resonant to intralayer K_{Mo}-K_{Mo} excitons. The momentum filter is placed at the K_{Mo} high-symmetry point (inset). The mean photoelectron energy shows an apparent upshift on the $<$ 200 fs scale as the excitonic energy relaxes. (**B**) Pump-probe delay evolution of EDCs including the K_W point and all three κ points. When applying a pump energy of 1.7 eV resonant to intralayer K_W-K_W excitons the mean photoelectron energy reduces as the exciton energy relaxes.

Microscopic modelling

In this section, we introduce the main concepts of the theoretical approach applied to calculate the dynamics in TMD bilayers. We start with the excitonic Hamilton operator

$$H = H_0 + H_T = \sum_{\mu,\mathbf{Q}} E^{\mu}_{\mathbf{Q}} X^{\mu\dagger}_{\mathbf{Q}} X^{\mu}_{\mathbf{Q}} + \sum_{\mu,\nu,\mathbf{Q}} \mathcal{T}_{\mu\nu} X^{\mu\dagger}_{\mathbf{Q}} X^{\nu}_{\mathbf{Q}} \tag{S5}$$

where we used the superindex $\mu = (n^{\mu}, \zeta^{\mu}_e, \zeta^{\mu}_h, L^{\mu}_e, L^{\mu}_h)$ to describe the exciton states, $E^{\mu}_{\mathbf{Q}} = E^{c}_{\zeta^{\mu}L^{\mu}_e} - E^{v}_{\zeta^{\mu}L^{\mu}_h} + E^{\mu}_{bind} + E^{\mu}_{\mathbf{Q},kin}$ are the excitonic energies, where E^{μ}_{bind} are obtained after solving a bilayer Wannier equation (*44, 64*), $E^{c/v}_{\zeta^{\mu}L^{\mu}_e}$ are the conduction and valence band energy and $E^{\mu}_{\mathbf{Q},kin} = \hbar^2\mathbf{Q}^2/(2M^{\mu})$ is the kinetic energy of the exciton with mass $M^{\mu} = (m^{\mu}_e + m^{\mu}_h)$. Moreover we introduced the excitonic tunneling between the TMD monolayers with the tunnelling matrix elements

$$\mathcal{T}_{\mu\nu} = (\delta_{L^{\mu}_h L^{\nu}_h}(1-\delta_{L^{\mu}_e L^{\nu}_e})\delta_{\zeta^{\mu}\zeta^{\nu}} T^{c}_{\mu_e,\nu_e} - \delta_{L^{\mu}_e L^{\nu}_e}(1-\delta_{L^{\mu}_h L^{\nu}_h})\delta_{\zeta^{\mu}\zeta^{\nu}} T^{v}_{\mu_h,\nu_h}) \sum_{\mathbf{k}} \psi^{\mu *}(\mathbf{k})\psi^{\nu}(\mathbf{k}), \tag{S6}$$

where ψ^{μ} is the excitonic wave function of the state μ defined over the relative momentum between electron and hole, $T^{\lambda}_{ij} = \langle \lambda i \mathbf{p}| H |\lambda j \mathbf{p}\rangle (1-\delta_{L_i L_j})\delta_{\zeta_i \zeta_j}$ are the electronic tunneling elements obtained by averaging DFT values of $MoSe_2$-WSe_2 and MoS_2-WS_2 heterostructures in (*65*). Diagonalizing Eq. S5 leads to a new set of hybrid excitonic energies $\mathcal{E}^{\eta}_{\mathbf{Q}}$ that are obtained by solving the hybrid eigenvalue equation (*64, 65*),

$$E^{\mu}_{\mathbf{Q}} c^{\eta}_{\mu}(\mathbf{Q}) + \sum_{\nu} \mathcal{T}_{\mu\nu} c^{\eta}_{\nu}(\mathbf{Q}) = \mathcal{E}^{\eta}_{\mathbf{Q}} c^{\eta}_{\mu}(\mathbf{Q}). \tag{S7}$$

Now, we can define a diagonal hybrid exciton Hamiltonian (*8, 42*)

$$H = \sum_{\eta} \mathcal{E}^{\eta}_{\mathbf{Q}} Y^{\eta\dagger}_{\mathbf{Q}} Y^{\eta}_{\mathbf{Q}} \tag{S8}$$

with the hybrid exciton annihilation/creation operators $Y^{\eta(\dagger)}_{\mathbf{Q}} = \sum_{\mu} c^{\eta}_{\mu}(\mathbf{Q}) X^{\mu(\dagger)}_{\mathbf{Q}}$. Evaluating the above eigenvalue equation, we predict the hybrid exciton energy landscape for the investigated WSe_2-MoS_2 heterostructure, see Fig. S7.

The hybrid exciton-phonon scattering plays a crucial role at the low excitation regime (*42, 66*). The corresponding Hamiltonian can be written as (*64*)

$$H_{Y-ph} = \sum_{j,\mathbf{Q},\mathbf{q},\eta,\xi} \tilde{\mathcal{D}}^{\xi\eta}_{j,\mathbf{q},\mathbf{Q}} Y^{\xi\dagger}_{\mathbf{Q}+\mathbf{q}} Y^{\eta}_{\mathbf{Q}} b_{j,\mathbf{q}} + h.c. \tag{S9}$$

with the hybrid exciton-phonon coupling $\tilde{\mathcal{D}}^{\xi\eta}_{j,\mathbf{q},\mathbf{Q}}$. The electron-phonon coupling matrix elements, single-particle energies and effective masses are taken from DFPT calculations (*43*). The excitation of the system through a laser pulse is described semi-classically via the minimal-coupling Hamiltonian that can be written as (*64*)

$$H_{Y-l} = \sum_{\sigma,\mathbf{Q},\eta} \mathbf{A} \cdot \tilde{\mathcal{M}}^{\eta}_{\sigma\mathbf{Q}} Y^{\eta}_{\mathbf{Q}_{\parallel}} + h.c. \tag{S10}$$

with hybrid exciton-light coupling $\tilde{\mathcal{M}}^{\eta}_{\sigma\mathbf{Q}}$. Details on the transformation and the definition of the hybrid interaction matrix elements and couplings are given in Ref. (*64, 65*).

Exploiting the Heisenberg equation of motion for the hybrid occupation $N^{\eta} = \langle Y^{\eta\dagger} Y^{\eta} \rangle$, including $H = H_Y + H_{Y-ph} + H_{Y-l}$, and truncating the Martin-Schwinger hierarchy using a second order Born-Markov approximation (*67–69*), separating coherent $P^{\eta}_{\mathbf{Q}} = \langle Y^{\eta\dagger}_{\mathbf{Q}} \rangle$ and incoherent hybrid populations $\delta N^{\eta}_{\mathbf{Q}} = \langle Y^{\eta\dagger}_{\mathbf{Q}} Y^{\eta}_{\mathbf{Q}} \rangle - \langle Y^{\eta\dagger}_{\mathbf{Q}} \rangle \langle Y^{\eta}_{\mathbf{Q}} \rangle = N^{\eta}_{\mathbf{Q}} - |P^{\eta}_{\mathbf{Q}}|^2$, leads to the coupled semiconductor Bloch equations

$$\begin{aligned} i\hbar\partial_t P^{\eta}_0 &= -(\mathcal{E}^{\eta}_0 + i\Gamma^{\eta}_0) P^{\eta}_0 - \tilde{\mathcal{M}}^{\eta}_0 \cdot \mathbf{A}(t) \\ \delta \dot{N}^{\eta}_{\mathbf{Q}} &= \sum_{\xi} W^{\xi\eta}_{\mathbf{0}\mathbf{Q}} |P^{\eta}_0|^2 + \sum_{\xi,\mathbf{Q}'} \left(W^{\xi\eta}_{\mathbf{Q}'\mathbf{Q}} \delta N^{\xi}_{\mathbf{Q}'} - W^{\eta\xi}_{\mathbf{Q}\mathbf{Q}'} \delta N^{\eta}_{\mathbf{Q}} \right) \end{aligned} \tag{S11}$$

with $W^{\eta\xi}_{\mathbf{Q}\mathbf{Q}'} = \frac{2\pi}{\hbar} \sum_{j,\pm} |\mathcal{D}^{\eta\xi}_{j,\mathbf{Q}'-\mathbf{Q}}|^2 \left(\frac{1}{2} \pm \frac{1}{2} + n^{ph}_{j,\mathbf{Q}'-\mathbf{Q}} \right) \delta \left(\mathcal{E}^{\xi}_{\mathbf{Q}'} - \mathcal{E}^{\eta}_{\mathbf{Q}} \mp \hbar\Omega_{j\mathbf{Q}'-\mathbf{Q}} \right)$ as the phonon mediated scattering tensor.

The large twist angle in the experiment gives rise to very short moire periods with a length scale comparable with the exciton Bohr radius. Therefore, a strong modification of the exciton center-of-mass motion, i.e. a moire-trapping of excitons is not expected (*70*) therefore, we neglect the twist angle dependence.

Resonant excitation of the K_{Mo}-K_{Mo} exciton leads also to a non-resonant excitation of the K_W-K_W state. The ratio in the exciton occupation of $N_{Mo}/N_W \simeq 5$ can be extracted from optical absorption coefficients (*41*). To model this effect in our simulations, we include one main pulse exciting the K_{Mo}-K_{Mo} state, and a secondary less intense pulse exciting the K_W-K_W state, imposing the same ratio of the coherent population as in the experiment.

The resulting evolution of exciton population for all contributing states is shown in Figure S8. Note that states with a valley degree of freedom cannot be distinguished in experiment. Therefore these states are summed up, e.g. K_{Mo}-K_{Mo} and K_{Mo}-K'_{Mo}.

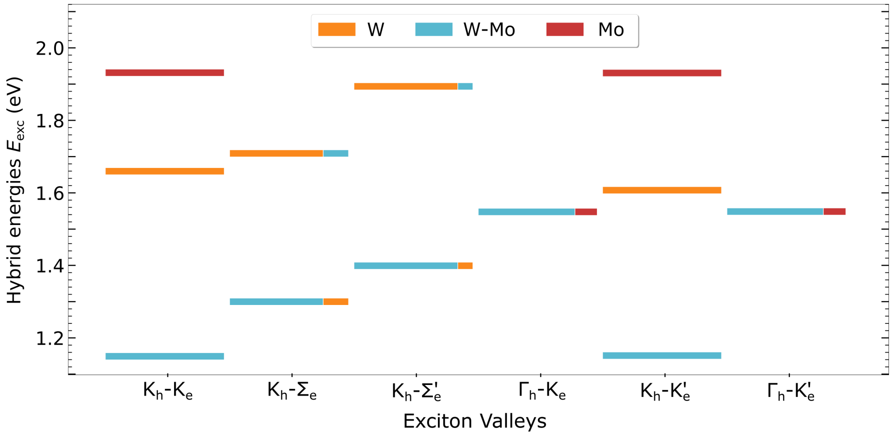

Figure S7: Hybrid-exciton energy landscape for the WSe_2/MoS_2 heterostructure. We use different colors for depicting the percentage of intralayer tungsten (W, orange), intralayer molybdenum (Mo, red) or interlayer (blue) exciton character of the corresponding states. Due to the strong tunneling experienced by electrons or holes, K_h- $\Sigma_e^{(\prime)}$ and Γ_h-$K_e^{(\prime)}$ states are strongly hybridized. Note that we plot only a selection of low-energy hybrid exciton states contributing directly to the relaxation dynamics.

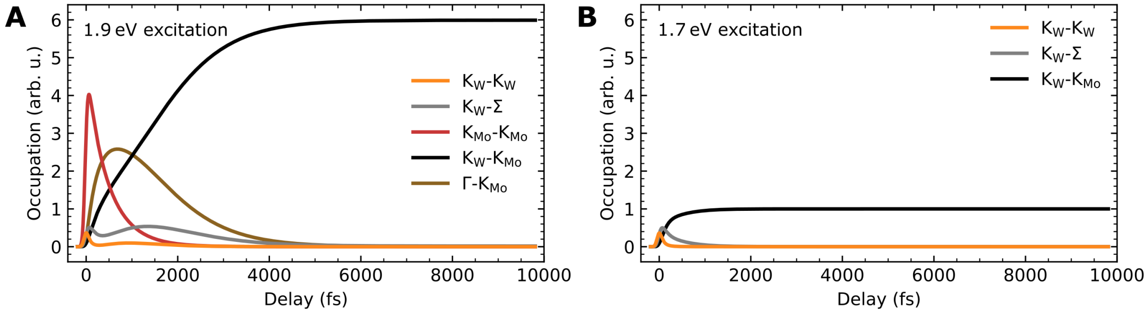

Figure S8: **Exciton occupation dynamics calculated for the electron- and the hole-transfer process.** Note that states with a valley degree of freedom cannot be distinguished in experiment. Therefore these states are summed up, e.g. K_{Mo}-K_{Mo} and K_{Mo}-K'_{Mo} excitons are termed K_{Mo}-K_{Mo} in the legend.

from \ to	K_W-K_W	K_W-K'_W	K_W-Σ	K_W-Σ'	K_W-K_{Mo}	K_W-K'_{Mo}	Γ-K_{Mo}	Γ-K'_{Mo}	K_{Mo}-K_{Mo}	K_{Mo}-K'_{Mo}
K_W-K_W	-	5.07	0.43	22.55	7E-04	1E-04	1E-04	0	0	0
K_W-K'_W	4E-09	-	18.95	1.04	8E-05	2E-03	0	3E-05	0	0
K_W-Σ	0	0	-	0	1.59	13.33	0	0	0	0
K_W-Σ'	0	0	142.77	-	5.43	0.83	0	0	0	0
K_W-K_{Mo}	0	0	0	0	-	0.47	0	0	0	0
K_W-K'_{Mo}	0	0	0	0	0	-	0	0	0	0
Γ-K_{Mo}	0	0	0	0	1.13	0	-	0	0	0
Γ-K'_{Mo}	0	0	0	0	0	1.00	0	-	0	0
K_{Mo}-K_{Mo}	3E-06	2E-07	5E-04	7E-03	2E-03	1E-04	1.62	0	-	0
K_{Mo}-K'_{Mo}	3E-07	2E-06	7E-03	6E-04	1E-04	2E-03	0	1.61	0.80	-

Table S1: Exciton-phonon scattering induced out-scattering rates for all initial exciton states resolved into all possible final states (ps^{-1}, for $Q = 0$). Out-scattering rates that are given with ”0” are fully suppressed due to energy conservation in the one phonon processes.

REFERENCES AND NOTES

1. G. Wang, A. Chernikov, M. M. Glazov, T. F. Heinz, X. Marie, T. Amand, B. Urbaszek, Colloquium: Excitons in atomically thin transition metal dichalcogenides. *Rev. Mod. Phys.* **90**, 021001 (2018).

2. A. Chernikov, T. C. Berkelbach, H. M. Hill, A. Rigosi, Y. Li, O. B. Aslan, D. R. Reichman, M. S. Hybertsen, T. F. Heinz, Exciton binding energy and nonhydrogenic rydberg series in monolayer WS_2. *Phys. Rev. Lett.* **113**, 076802 (2014).

3. K. He, N. Kumar, L. Zhao, Z. Wang, K. F. Mak, H. Zhao, J. Shan, Tightly bound excitons in monolayer WSe_2. *Phys. Rev. Lett.* **113**, 026803 (2014).

4. C.-H. Lee, G.-H. Lee, A. M. van der Zande, W. Chen, Y. Li, M. Han, X. Cui, G. Arefe, C. Nuckolls, T. F. Heinz, J. Guo, J. Hone, P. Kim, Atomically thin p-n junctions with van der Waals heterointerfaces. *Nat. Nanotechnol.* **9**, 676–681 (2014).

5. X. Hong, J. Kim, S.-F. Shi, Y. Zhang, C. Jin, Y. Sun, S. Tongay, J. Wu, Y. Zhang, F. Wang, Ultrafast charge transfer in atomically thin MoS_2/WS_2 heterostructures. *Nat. Nanotechnol.* **9**, 682–686 (2014).

6. F. Ceballos, M. Z. Bellus, H.-Y. Chiu, H. Zhao, Ultrafast charge separation and indirect exciton formation in a MoS_2-$MoSe_2$ van der waals heterostructure. *ACS Nano* **8**, 12717–12724 (2014).

7. P. Merkl, F. Mooshammer, P. Steinleitner, A. Girnghuber, K. Q. Lin, P. Nagler, J. Holler, C. Schüller, J. M. Lupton, T. Korn, S. Ovesen, S. Brem, E. Malic, R. Huber, Ultrafast transition between exciton phases in van der Waals heterostructures. *Nat. Mater.* **18**, 691–696 (2019).

8. D. Schmitt, J. P. Bange, W. Bennecke, A. AlMutairi, G. Meneghini, K. Watanabe, T. Taniguchi, D. Steil, D. R. Luke, R. T. Weitz, S. Steil, G. S. M. Jansen, S. Brem, E. Malic, S. Hofmann, M. Reutzel, S. Mathias, Formation of moiré interlayer excitons in space and time. *Nature* **608**, 499–503 (2022).

9. K. L. Seyler, P. Rivera, H. Yu, N. P. Wilson, E. L. Ray, D. G. Mandrus, J. Yan, W. Yao, X. Xu, Signatures of moiré-trapped valley excitons in $MoSe_2/WSe_2$ heterobilayers. *Nature* **567**, 66–70 (2019).

10. E. M. Alexeev, D. A. Ruiz-Tijerina, M. Danovich, M. J. Hamer, D. J. Terry, P. K. Nayak, S. Ahn, S. Pak, J. Lee, J. I. Sohn, M. R. Molas, M. Koperski, K. Watanabe, T. Taniguchi, K. S. Novoselov, R. V. Gorbachev, H. S. Shin, V. I. Fal'ko, A. I. Tartakovskii, Resonantly hybridized excitons in moiré superlattices in van der Waals heterostructures. *Nature* **567**, 81–86 (2019).

11. K. Tran, G. Moody, F. Wu, X. Lu, J. Choi, K. Kim, A. Rai, D. A. Sanchez, J. Quan, A. Singh, J. Embley, A. Zepeda, M. Campbell, T. Autry, T. Taniguchi, K. Watanabe, N. Lu, S. K. Banerjee, K. L. Silverman, S. Kim, E. Tutuc, L. Yang, A. H. MacDonald, X. Li, Evidence for moiré excitons in van der Waals heterostructures. *Nature* **567**, 71–75 (2019). 12. O. Karni, E. Barré, V. Pareek, J. D. Georgaras, M. K. L. Man, C. Sahoo, D. R. Bacon, X. Zhu, H. B. Ribeiro, A. L. O'Beirne, J. Hu, A. Al-Mahboob, M. M. M. Abdelrasoul, N. S. Chan, A. Karmakar, A. J. Winchester, B. Kim, K. Watanabe, T. Taniguchi, K. Barmak, J. Madéo, F. H. da Jornada, T. F. Heinz, K. M. Dani, Structure of the moiré exciton captured by imaging its electron and hole. *Nature* **603**, 247–252 (2022).

13. L. Ma, P. X. Nguyen, Z. Wang, Y. Zeng, K. Watanabe, T. Taniguchi, A. H. MacDonald, K. F. Mak, J. Shan, Strongly correlated excitonic insulator in atomic double layers. *Nature* **598**, 585–589 (2021).

14. Z. Zhang, E. C. Regan, D. Wang, W. Zhao, S. Wang, M. Sayyad, K. Yumigeta, K. Watanabe, T. Taniguchi, S. Tongay, M. Crommie, A. Zettl, M. P. Zaletel, F. Wang, Correlated interlayer exciton insulator in heterostructures of monolayer WSe_2 and moiré WS_2/WSe_2. *Nat. Phys.* **18**, 1214–1220 (2022).

15. Y. Slobodkin, Y. Mazuz-Harpaz, S. Refaely-Abramson, S. Gazit, H. Steinberg, R. Rapaport, Quantum phase transitions of trilayer excitons in atomically thin heterostructures. *Phys. Rev. Lett.* **125**, 255301 (2020).

16. L. Sigl, F. Sigger, F. Kronowetter, J. Kiemle, J. Klein, K. Watanabe, T. Taniguchi, J. J. Finley, U. Wurstbauer, A. W. Holleitner, Signatures of a degenerate many-body state of interlayer excitons in a van der waals heterostack. *Phys. Rev. Res.* **2**, 042044 (2020).
17. Z. Wang, D. A. Rhodes, K. Watanabe, T. Taniguchi, J. C. Hone, J. Shan, K. F. Mak, Evidence of high-temperature exciton condensation in two-dimensional atomic double layers. *Nature* **574**, 76–80 (2019).
18. C. Jin, E. Y. Ma, O. Karni, E. C. Regan, F. Wang, T. F. Heinz, Ultrafast dynamics in van der Waals heterostructures. *Nat. Nanotechnol.* **13**, 994–1003 (2018).
19. R. Perea-Causin, D. Erkensten, J. M. Fitzgerald, J. J. P. Thompson, R. Rosati, S. Brem, E. Malic, Exciton optics, dynamics, and transport in atomically thin semiconductors. *APL Mater.* **10**, 100701 (2022).
20. C. Poellmann, P. Steinleitner, U. Leierseder, P. Nagler, G. Plechinger, M. Porer, R. Bratschitsch, C. Schüller, T. Korn, R. Huber, Resonant internal quantum transitions and femtosecond radiative decay of excitons in monolayer WSe_2. *Nat. Mater.* **14**, 889–893 (2015).
21. E. Malic, M. Selig, M. Feierabend, S. Brem, D. Christiansen, F. Wendler, A. Knorr, G. Berghäuser, Dark excitons in transition metal dichalcogenides. *Phys. Rev. Mater.* **2**, 014002 (2018).
22. T. Mueller, E. Malic, Exciton physics and device application of two-dimensional transition metal dichalcogenide semiconductors. *NPJ 2D Mater. Appl.* **2**, 29 (2018).
23. J. Madéo, M. K. L. Man, C. Sahoo, M. Campbell, V. Pareek, E. L. Wong, A. Al-Mahboob, N. S. Chan, A. Karmakar, B. M. K. Mariserla, X. Li, T. F. Heinz, T. Cao, K. M. Dani, Directly visualizing the momentum-forbidden dark excitons and their dynamics in atomically thin semiconductors. *Science* **370**, 1199–1204 (2020).
24. R. Wallauer, R. Perea-Causin, L. Münster, S. Zajusch, S. Brem, J. Güdde, K. Tanimura, K.-Q. Lin, R. Huber, E. Malic, U. Höfer, Momentum-resolved observation of exciton formation dynamics in monolayer WS_2. *Nano Lett.* **21**, 5867–5873 (2021).

25. A. Kunin, S. Chernov, J. Bakalis, Z. Li, S. Cheng, Z. H. Withers, M. G. White, G. Schönhense, X. Du, R. K. Kawakami, T. K. Allison, Momentum-resolved exciton coupling and valley polarization dynamics in monolayer WS_2. *Phys. Rev. Lett.* **130**, 046202 (2023).

26. D. Schmitt, J. P. Bange, W. Bennecke, G. Meneghini, A. AlMutairi, M. Merboldt, J. Pöhls, K. Watanabe, T. Taniguchi, S. Steil, D. Steil, R. T. Weitz, S. Hofmann, S. Brem, G. S. Matthijs Jansen, E. Malic, S. Mathias, M. Reutzel, Ultrafast nano-imaging of dark excitons. arXiv:2305.18908 (2023).

27. J. P. Bange, P. Werner, D. Schmitt, W. Bennecke, G. Meneghini, A. AlMutairi, M. Merboldt, K. Watanabe, T. Taniguchi, S. Steil, D. Steil, R. T. Weitz, S. Hofmann, G. S. M. Jansen, S. Brem, E. Malic, M. Reutzel, S. Mathias, Ultrafast dynamics of bright and dark excitons in monolayer WSe_2 and heterobilayer WSe_2/MoS_2. *2D Mater.* **10**, 035039 (2023).

28. E. Perfetto, D. Sangalli, A. Marini, G. Stefanucci, First-principles approach to excitons in time-resolved and angle-resolved photoemission spectra. *Phys. Rev. B* **94**, 245303 (2016).

29. A. Rustagi, A. F. Kemper, Photoemission signature of excitons. *Phys. Rev. B* **97**, 235310 (2018).

30. A. Steinhoff, M. Florian, M. Rösner, G. Schönhoff, T. O. Wehling, F. Jahnke, Exciton fission in monolayer transition metal dichalcogenide semiconductors. *Nat. Commun.* **8**, 1166 (2017).

31. D. Christiansen, M. Selig, E. Malic, R. Ernstorfer, A. Knorr, Theory of exciton dynamics in time-resolved ARPES: Intra- and intervalley scattering in two-dimensional semiconductors. *Phys. Rev. B* **100**, 205401 (2019).

32. S. Dong, M. Puppin, T. Pincelli, S. Beaulieu, D. Christiansen, H. Hübener, C. W. Nicholson, R. P. Xian, M. Dendzik, Y. Deng, Y. W. Windsor, M. Selig, E. Malic, A. Rubio, A. Knorr, M. Wolf, L. Rettig, R. Ernstorfer, Direct measurement of key exciton properties: Energy, dynamics, and spatial distribution of the wave function. *Nat. Sci.* **1**, e10010 (2021).

33. M. K. L. Man, J. Madéo, C. Sahoo, K. Xie, M. Campbell, V. Pareek, A. Karmakar, E. L. Wong, A. Al-Mahboob, N. S. Chan, D. R. Bacon, X. Zhu, M. M. M. Abdelrasoul, X. Li, T. F. Heinz, F. H. da

Jornada, T. Cao, K. M. Dani, Experimental measurement of the intrinsic excitonic wave function. *Sci. Adv.* **7**, eabg0192 (2021).

34. H. Zhu, J. Wang, Z. Gong, Y. D. Kim, J. Hone, X. Y. Zhu, Interfacial charge transfer circumventing momentum mismatch at two-dimensional van der Waals heterojunctions. *Nano Lett.* **17**, 3591–3598 (2017).

35. J. E. Zimmermann, M. Axt, F. Mooshammer, P. Nagler, C. Schüller, T. Korn, U. Höfer, G. Mette, Ultrafast charge-transfer dynamics in twisted MoS_2/WSe_2 heterostructures. *ACS Nano* **15**, 14725–14731 (2021).

36. V. R. Policht, M. Russo, F. Liu, C. Trovatello, M. Maiuri, Y. Bai, X. Zhu, S. Dal Conte, G. Cerullo, Dissecting interlayer hole and electron transfer in transition metal dichalcogenide heterostructures via two-dimensional electronic spectroscopy. *Nano Lett.* **21**, 4738–4743 (2021).

37. Z. Wang, P. Altmann, C. Gadermaier, Y. Yang, W. Li, L. Ghirardini, C. Trovatello, M. Finazzi, L. Duò, M. Celebrano, R. Long, D. Akinwande, O. V. Prezhdo, G. Cerullo, S. Dal Conte, Phonon-mediated interlayer charge separation and recombination in a $MoSe_2/WSe_2$ heterostructure. *Nano Lett.* **21**, 2165–2173 (2021).

38. G. Meneghini, M. Reutzel, S. Mathias, S. Brem, E. Malic, Hybrid exciton signatures in ARPES spectra of van der waals materials. *ACS Photonics* **10**, 3570–3575 (2023).

39. M. Keunecke, C. Möller, D. Schmitt, H. Nolte, G. S. M. Jansen, M. Reutzel, M. Gutberlet, G. Halasi, D. Steil, S. Steil, S. Mathias, Time-resolved momentum microscopy with a 1 MHz high-harmonic extreme ultraviolet beamline. *Rev. Sci. Instrum.* **91**, 063905 (2020).

40. M. Keunecke, M. Reutzel, D. Schmitt, A. Osterkorn, T. A. Mishra, C. Möller, W. Bennecke, G. S. M. Jansen, D. Steil, S. R. Manmana, S. Steil, S. Kehrein, S. Mathias, Electromagnetic dressing of the electron energy spectrum of Au(111) at high momenta. *Phys. Rev. B* **102**, 161403 (2020).

41. Y. Li, A. Chernikov, X. Zhang, A. Rigosi, H. M. Hill, A. M. van der Zande, D. A. Chenet, E.-M. Shih, J. Hone, T. F. Heinz, Measurement of the optical dielectric function of monolayer transition-metal dichalcogenides: MoS_2, $MoSe_2$, WS_2, and WSe_2. *Phys. Rev. B* **90**, 205422 (2014).
42. G. Meneghini, S. Brem, E. Malic, Ultrafast phonon-driven charge transfer in van der Waals heterostructures. *Nat. Sci.* **2**, e20220014 (2022).
43. Z. Jin, X. Li, J. T. Mullen, K. W. Kim, Intrinsic transport properties of electrons and holes in monolayer transition-metal dichalcogenides. *Phys. Rev. B* **90**, 045422 (2014).
44. S. Ovesen, S. Brem, C. Linderälv, M. Kuisma, T. Korn, P. Erhart, M. Selig, E. Malic, Interlayer exciton dynamics in van der Waals heterostructures. *Commun. Phys.* **2**, 23 (2019).
45. M. Selig, G. Berghäuser, M. Richter, R. Bratschitsch, A. Knorr, E. Malic, Dark and bright exciton formation, thermalization, and photoluminescence in monolayer transition metal dichalcogenides. *2D Mater.* **5**, 035017 (2018).
46. A. Raja, M. Selig, G. Berghäuser, J. Yu, H. M. Hill, A. F. Rigosi, L. E. Brus, A. Knorr, T. F. Heinz, E. Malic, A. Chernikov, Enhancement of exciton-phonon scattering from monolayer to bilayer WS_2. *Nano Lett.* **18**, 6135–6143 (2018).
47. J. Lindlau, M. Selig, A. Neumann, L. Colombier, J. Förste, V. Funk, M. Förg, J. Kim, G. Berghäuser, T. Taniguchi, K. Watanabe, F. Wang, E. Malic, A. Högele, The role of momentum-dark excitons in the elementary optical response of bilayer WSe_2. *Nat. Commun.* **9**, 2586 (2018).
48. D. Sun, Y. Rao, G. A. Reider, G. Chen, Y. You, L. Brézin, A. R. Harutyunyan, T. F. Heinz, Observation of rapid exciton-exciton annihilation in monolayer molybdenum disulfide. *Nano Lett.* **14**, 5625–5629 (2014).
49. D. Erkensten, S. Brem, K. Wagner, R. Gillen, R. Perea-Causín, J. D. Ziegler, T. Taniguchi, K. Watanabe, J. Maultzsch, A. Chernikov, E. Malic, Dark exciton-exciton annihilation in monolayer WSe_2. *Phys. Rev. B* **104**, L241406 (2021).

50. A. Damascelli, Z. Hussain, Z.-X. Shen, Angle-resolved photoemission studies of the cuprate superconductors. *Rev. Mod. Phys.* **75**, 473–541 (2003).

51. M. Bauer, A. Marienfeld, M. Aeschlimann, Hot electron lifetimes in metals probed by time-resolved two-photon photoemission, *Prog. Surf. Sci.* **90**, 319–376 (2015).

52. S. Mathias, S. Eich, J. Urbancic, S. Michael, A. V. Carr, S. Emmerich, A. Stange, T. Popmintchev, T. Rohwer, M. Wiesenmayer, A. Ruffing, S. Jakobs, S. Hellmann, P. Matyba, C. Chen, L. Kipp, M. Bauer, H. C. Kapteyn, H. C. Schneider, K. Rossnagel, M. M. Murnane, M. Aeschlimann, Self-amplified photo-induced gap quenching in a correlated electron material. *Nat. Commun.* **7**, 12902 (2016).

53. A. Chernikov, C. Ruppert, H. M. Hill, A. F. Rigosi, T. F. Heinz, Population inversion and giant bandgap renormalization in atomically thin WS_2 layers. *Nat. Photonics* **9**, 466–470 (2015).

54. F. Liu, M. E. Ziffer, K. R. Hansen, J. Wang, X. Zhu, Direct determination of band-gap renormalization in the photoexcited monolayer MoS_2. *Phys. Rev. Lett.* **122**, 246803 (2019).

55. M. Weinelt, M. Kutschera, T. Fauster, M. Rohlfing, Dynamics of exciton formation at the Si(100) c(4 x 2) surface. *Phys. Rev. Lett.* **92**, 126801 (2004).

56. O. Karni, E. Barré, S. C. Lau, R. Gillen, E. Y. Ma, B. Kim, K. Watanabe, T. Taniguchi, J. Maultzsch, K. Barmak, R. H. Page, T. F. Heinz, Infrared interlayer exciton emission in MoS−2/WSe_2 heterostructures. *Phys. Rev. Lett.* **123**, 247402 (2019).

57. J. Kunstmann, F. Mooshammer, P. Nagler, A. Chaves, F. Stein, N. Paradiso, G. Plechinger, C. Strunk, C. Schüller, G. Seifert, D. R. Reichman, T. Korn, Momentum-space indirect interlayer excitons in transition-metal dichalcogenide van der Waals heterostructures. *Nat. Phys.* **14**, 801–805 (2018).

58. K. Medjanik, O. Fedchenko, S. Chernov, D. Kutnyakhov, M. Ellguth, A. Oelsner, B. Schönhense, T. R. F. Peixoto, P. Lutz, C.-H. Min, F. Reinert, S. Däster, Y. Acremann, J. Viefhaus, W. Wurth, H.

J. Elmers, G. Schönhense, Direct 3D mapping of the Fermi surface and Fermi velocity. *Nat. Mater.* **16**, 615–621 (2017).

59. B. Krömker, M. Escher, D. Funnemann, D. Hartung, H. Engelhard, J. Kirschner, Development of a momentum microscope for time resolved band structure imaging. *Rev. Sci. Instrum.* **79**, 053702–7 (2008).

60. M. Düvel, M. Merboldt, J. P. Bange, H. Strauch, M. Stellbrink, K. Pierz, H. W. Schumacher, D. Momeni, D. Steil, G. S. M. Jansen, S. Steil, D. Novko, S. Mathias, M. Reutzel, Far-from-equilibrium electron-phonon interactions in optically excited graphene. *Nano Lett.* **22**, 4897–4904 (2022).

61. G. Saathoff, L. Miaja-Avila, M. Aeschlimann, M. M. Murnane, H. C. Kapteyn, Laser-assisted photoemission from surfaces, *Phys. Rev. A* **77**, 022903 (2008).

62. T. Taniguchi, K. Watanabe, Synthesis of high-purity boron nitride single crystals under high pressure by using Ba-BN solvent. *J. Cryst. Growth* **303**, 525–529 (2007).

63. G. Schönhense, D. Kutnyakhov, F. Pressacco, M. Heber, N. Wind, S. Y. Agustsson, S. Babenkov, D. Vasilyev, O. Fedchenko, S. Chernov, L. Rettig, B. Schönhense, L. Wenthaus, G. Brenner, S. Dziarzhytski, S. Palutke, S. K. Mahatha, N. Schirmel, H. Redlin, B. Manschwetus, I. Hartl, Y. Matveyev, A. Gloskovskii, C. Schlueter, V. Shokeen, H. Duerr, T. K. Allison, M. Beye, K. Rossnagel, H. J. Elmers, K. Medjanik, Suppression of the vacuum space-charge effect in fs-photoemission by a retarding electrostatic front lens. *Rev. Sci. Instrum.* **92**, 053703 (2021).

64. S. Brem, K.-Q. Lin, R. Gillen, J. M. Bauer, J. Maultzsch, J. M. Lupton, E. Malic, Hybridized intervalley moiré excitons and flat bands in twisted WSe_2 bilayers. *Nanoscale* **12**, 11088–11094 (2020).

65. J. Hagel, S. Brem, C. Linderälv, P. Erhart, E. Malic, Exciton landscape in van der Waals heterostructures. *Phys. Rev. Res.* **3**, 043217 (2021).

66. S. Brem, M. Selig, G. Berghaeuser, E. Malic, Exciton relaxation cascade in two-dimensional transition metal dichalcogenides. *Sci. Rep.* **8**, 8238 (2018).

67. M. Kira, S. W. Koch, Many-body correlations and excitonic effects in semiconductor spectroscopy. *Prog. Quantum Electron.* **30**, 155–296 (2006).

68. H. Haug, S. W. Koch, *Quantum Theory of the Optical and Electronic Properties of Semiconductors* (World Scientific Publishing Company, 2009).

69. E. Malic, A. Knorr, *Graphene and Carbon Nanotubes: Ultrafast Optics and Relaxation Dynamics* (John Wiley & Sons, 2013).

70. S. Brem, C. Linderälv, P. Erhart, E. Malic, Tunable phases of moiré excitons in van der Waals heterostructures. *Nano Lett.* **20**, 8534–8540 (2020).
